# Human Autoantibodies Against N-Methyl-D-Aspartate Receptor Modestly Alter Dopamine D1 Receptor Surface Dynamics

**DOI:** 10.3389/fpsyt.2019.00670

**Published:** 2019-09-13

**Authors:** Hélène Gréa, Delphine Bouchet, Véronique Rogemond, Nora Hamdani, Emmanuel Le Guen, Ryad Tamouza, Estelle Darrau, Christine Passerieux, Jérôme Honnorat, Marion Leboyer, Laurent Groc

**Affiliations:** ^1^ Université de Bordeaux, Interdisciplinary Institute for Neuroscience, UMR 5297, Bordeaux, France; ^2^CNRS, IINS UMR 5297, Bordeaux, France; ^3^ NeuroMyoGene Institute, INSERM U1217/CNRS UMR 5310, Lyon, France; ^4^French Reference Center of Paraneoplastic Neurological Syndrome, Hospices Civils de Lyon, Hôpital Neurologique, Lyon, France; ^5^ Université de Lyon - Université Claude Bernard Lyon 1, Lyon, France; ^6^Université Paris Est Créteil, Psychiatry Department, Groupe Hospitalier Universitaire Henri Mondor, AP-HP, DHU PePSY, Créteil, France; ^7^Université Paris Est Créteil, Life Science and Health Department, INSERM IMRB U955, Créteil, France; ^8^INSERM, IMRB U955, Translational Psychiatry laboratory, Créteil, France; ^9^FondaMental foundation, Créteil, France; ^10^Université de Versaillles Saint Quentin en Yvelines, HandiRESP laboratory (EA4047), Health Science Department Simone Veil, Montigny le Bretonneux, France; ^11^Versailles Hospital, Department of Adult Psychiatry, Le Chesnay, France

**Keywords:** autoimmunity, encephalitis, schizophrenia, autoimmune psychosis, dopamine, single molecule imaging, hippocampal neurons

## Abstract

Circulating autoantibodies directed against extracellular domains of the glutamatergic N-methyl-D-aspartate receptors (NMDAR-Ab) elicit psychotic symptoms in humans and behavioral deficits in animal models. Recent advances suggest that NMDAR-Ab exert their pathogenic action by altering the trafficking of NMDAR, which results in a synaptic NMDAR hypofunction consistent with the consensual glutamatergic hypothesis of psychotic disorders. Yet, dysfunction in the dopaminergic signaling is also proposed to be at the core of psychotic disorders. Since NMDAR and dopamine D1 receptors (D1R) form membrane signaling complexes, we investigated whether NMDAR-Ab purified from patients with NMDAR-encephalitis or schizophrenia impaired D1R surface dynamics. As previous data demonstrated that NMDAR-Ab, at least from NMDAR-encephalitis patients, mainly bind to hippocampal NMDAR, we used single nanoparticle imaging to track D1R specifically at the surface of hippocampal neurons that were exposed to either purified G type immunoglobulins (IgGs) from NMDAR-Ab seropositive patients suffering from NMDAR-encephalitis or schizophrenia, or control IgGs from healthy NMDAR-Ab seropositive or seronegative subjects. We report that overnight incubation with NMDAR-Ab from patients, but not from healthy carriers, decreased the surface dynamics of D1R compared with NMDAR-Ab seronegative IgGs. This decrease was abolished, and even reversed, in D1R mutant that cannot physically interact with NMDAR. Overall, our data indicate that NMDAR-Ab from patients with psychotic symptoms alter the trafficking of D1R, likely through the surface crosstalk between NMDAR and D1R.

## Introduction

Psychotic disorders, such as schizophrenia, are major mental illnesses with multiple etiologies. During the past decades, accumulating evidence suggests that dysregulations of the immune system, such as the presence of autoantibodies directed against neurotransmitter receptors, play a major role in psychosis (1–5), paving the way for the recognition of an autoimmune psychosis subclass (6). The discovery of the well-characterized N-methyl-D-aspartate receptor (NMDAR)-encephalitis demonstrated that circulating autoantibodies targeting the NMDAR (i.e., NMDAR-Ab) play an instrumental and pathogenic role (7). Indeed, the presence of NMDAR-Ab in the sera of NMDAR-encephalitis patients correlates, in a titer-dependent manner, with psychotic-like symptoms that appear at early stage of the illness. At the molecular level, autoantibodies from NMDAR-encephalitis patients laterally displace synaptic NMDAR toward the extrasynaptic membrane, in which they are physically cross-linked and internalized, leading to the downregulation of NMDAR-mediated signaling (8, 9). Recently, NMDAR-Ab have also been found in the sera of a significant proportion of patients diagnosed with schizophrenia (10) but also in a very few healthy carriers (11). Similarly to NMDAR-Ab from encephalitis patients, NMDAR-Ab from psychotic patients, but not from healthy subjects, laterally displace synaptic NMDAR toward the extrasynaptic membrane (12). Thus, different molecular cascades are triggered by NMDAR-Ab from different origins, calling for caution in generalizing the impact of these autoantibodies. Although the identification of NMDAR-Ab has further fueled the hypothesis of a NMDAR hypofunction in psychosis (13), gold-standard treatments of psychotic disorders remain composed of antagonists of the dopamine receptors and other monoamine systems (e.g., serotonin) (14–16). Understanding how the glutamatergic and dopaminergic systems influence each other and likely participate to the etiology of psychotic disorders is still obviously a major challenge in the field of psychiatry. The fact that NMDAR physically interacts with dopamine receptors [e.g., dopamine D1 receptor (D1R)] in an agonist-dependent manner indicates that, already at the plasma membrane level, a functional interplay between dopaminergic and NMDAR signaling exists (17). We here hypothesize that the altered surface trafficking of NMDAR triggered by NMDAR-Ab from patients with NMDAR-encephalitis or schizophrenia, but not from healthy carriers, could then modify the surface dynamics of D1R. As NMDAR-Ab from patients with NMDAR-encephalitis mainly bind to NMDAR in the hippocampus (18), we investigated the molecular impact of NMDAR-Ab on D1R surface dynamics in a model of cultured hippocampal neurons. A former investigation revealed that a short incubation (2 h) of hippocampal cell networks with NMDAR-Ab from encephalitis patients did not alter D1R surface trafficking (8). Herein, we used a single molecule-based imaging approach to assess the D1R surface dynamics in hippocampal neurons exposed for a longer incubation period (overnight) to NMDAR-Ab [purified G type immunoglobulins (IgGs)] from either healthy seropositive carriers (Healthy+), patients with NMDAR-encephalitis (Enceph), or schizophrenia (SCZ+), or seronegative matched-healthy subjects (Healthy-). In order to assess if the expected alteration of D1R surface dynamics is a direct consequence of the physical interaction between D1R and NMDAR-Ab-targeted NMDAR, we investigated the surface diffusion of a truncated exogenous D1R, which prevents its physical interaction with NMDAR, expressed in hippocampal neurons exposed to purified IgGs from a patient with schizophrenia compared with an healthy seronegative subject.

## Methods

### Participants

Five patients with NMDAR-encephalitis (Enceph) and three patients with schizophrenia (SCZ+) (Diagnostic and Statistical Manual of Mental Disorders, 5th Edition criteria), all seropositive for NMDAR-Ab, were included in this study after approval by a French ethical committee (**Table 1**). Patients with NMDAR-encephalitis had no psychiatric history and were recruited from a cohort of 400 NMDAR-encephalitis patients (French National Reference Centre for Paraneoplastic Neurological Syndromes and Autoimmune Encephalitis, Bron, France). Patients with schizophrenia were recruited after admission to two university-affiliated psychiatric departments (Mondor Hospital, Créteil, University of Paris-Est, and Fernand Widal Hospital, Paris, University of Diderot, Paris), and any history of stroke, multiple sclerosis, epilepsy, or encephalitis constituted exclusion criteria. The clinical state of both type of patients could assure their total capacity to understand the aims and the procedures of the study and finally to express their will to participate in a written informed consent. Two seropositive (Healthy+) and five seronegative (Healthy-) for NMDAR-Ab healthy matched for age, gender, and years of education subjects with no personal or familial history of psychosis were included in the study as controls.

**Table 1:** Clinical features of seropositive for NMDAR-Ab patients with either NMDAR-encephalitis (Enceph) or schizophrenia (SCZ+).

	Age at onset/sex	Clinical symptoms*	Treatments			
Patients with NMDAR-encephalitis (Enceph)				ICU	Cancer	Outcome
1	18/F	Hallucinations, abnormal behavior, abnormal movements	PE, C, IvIg, Cyclophosphamide	No	No	Cured after 24 months
2	29/F	Hallucinations, abnormal behavior, abnormal movements, epilepsy	C, IvIg, Cyclophosphamide Rituximab Micophenolate mophetyl	Yes 10 days	Ovarian teratoma	Cured after 24 months
3	21/F**	Hallucinations, abnormal behavior, abnormal movements	Cyclophosphamide Rituximab Micophenolate mophetyl	No	No	Cured after 24 months
4	18/F**	Hallucinations, epilepsy, abnormal behavior, abnormal movements	C, IvIg, Azathioprine	No	No	Cured after 18 months
5	22/F**	Hallucinations, abnormal behavior, abnormal movements, epilepsy, dysautonomia	C, IvIg, Cyclophosphamide Micophenolate mophetyl	Yes 1 month	No	Cured after 9 months
Patients with schizophrenia (SCZ+)				PANSS Total (>60) Positive scale score Negative scale score	MRI	Other medical history
1	22/M	Blunted affects Disorganization Suicidal thoughts	Risperidone (4 mg/day) Cyamemazine (75 mg/day) Oxazepam (30 mg/day) Duloxetine (60 mg/day)	66 Positive scale : 7 Negative scale : 26	normal	Dyslipidemia Type 2 diabetes mellitus
2	34/F	Cognitive impairment Delusions Attention deficits Blunted affects	Aripiprazole (30 mg/day) Escitaloprame (10 mg/day) Hydroxyzine (300 mg/day)	132 Positive scale : 26 Negative scale : 42	none	none
3	25/M	Blunted affects Cognitive impairment Delusions Disorganization	Clozapine (100 mg/day) Loxapine (150 mg/day)	76 Positive scale : 14 Negative scale : 19	normal	Epilepsia Head trauma Hepatic colic

ICU, intensive care unit; C, corticosteroids; PE, plasma exchange; IvIg, intravenous immunoglobulines. PANSS total, Positive and Negative Syndrome Scale total score. *Symptoms at presentation for patients with NMDAR-encephalitis and residual/active symptoms for patients with schizophrenia. **Purified G type immunoglobulins (IgGs) from patients with NMDAR-encephalitis #3, #4, and #5 were available pooled together.

### Purified Type G Immunoglobulins From Participants

All experiments were conducted using purified IgGs containing (Healthy+, SCZ+, Enceph) or not containing (Healthy-) NMDAR-Ab from subjects’ sera. In all experiments, purified IgGs were used from separate individuals, except for three out of the five NMDAR-encephalitis patients and three out of the five healthy seronegative subjects for whom pooled IgGs were available.

### Detection of NMDAR Autoantibodies in Participants’ Sera

For patients with NMDAR-encephalitis or schizophrenia, sera were collected at symptom presentation, before any treatment and stored at -80°C. The presence of NMDAR-Ab in sera of either patients or control subjects was assessed using a classic cell-based assay. Briefly, exogenous NMDAR were ectopically expressed in human embryonic kidney cells (HEK) 293 transfected with GluN1-NMDAR subunit fused to the green fluorescent protein (GFP) along with GluN2B-NMDAR subunit to promote the insertion of functional NMDAR at the cell surface. After a 48-h expression period, live HEK cells were incubated with subjects’ sera (3 h, 1/20 in saturation buffer). Then, fixed HEK cells were incubated with anti-human IgG coupled to Alexa 555. Using a fluorescence microscope, the observation of an overlap of both green and red staining led to the assessment of the subject seropositivity for NMDAR-Ab.

### Primary Cell Culture and Single Quantum Dot Tracking

**Figure 1:**
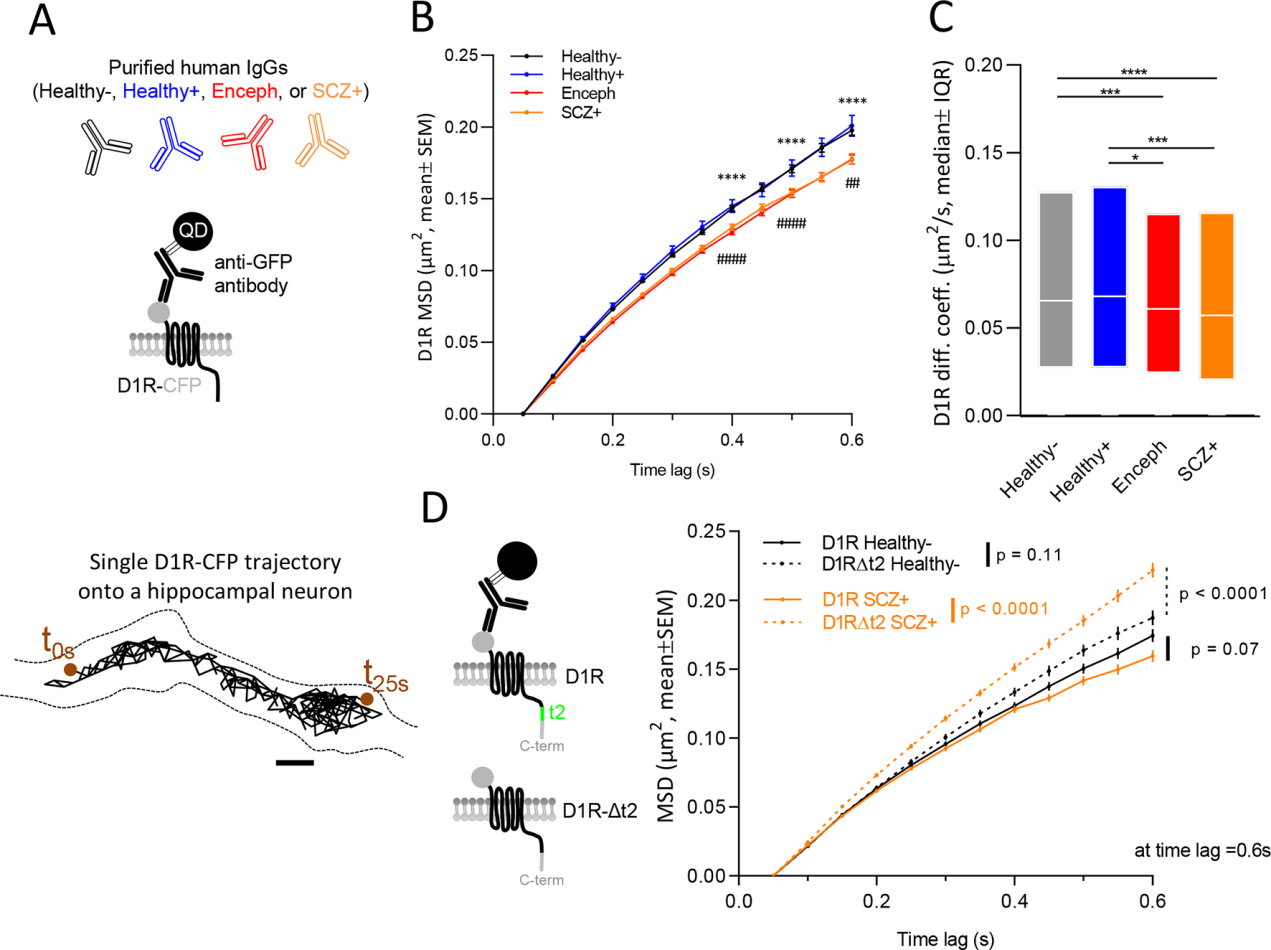
NMDAR-Ab from patients with NMDAR-encephalitis (Enceph) or schizophrenia (SCZ+), but not from healthy carriers (Healthy+), alter the surface dynamics of D1R compared with healthy seronegative subjects (Healthy-). **(A)** Schematic representation of the experimental design. Before tracking, D1R-CFP-antiGFPAb-QD complexes, hippocampal cultures (12 to 15 days *in vitro*) were incubated overnight with different purified type G immunoglobulins (IgGs, 5 µm/ml) samples from either patients with NMDAR-encephalitis (Enceph), schizophrenia (SCZ+), healthy carriers (Healthy+), or healthy seronegative subjects (Healthy-) (top panel). Representative trajectory of a single D1R-CFP-antiGFPAb-QD complex (500 frames, 50 ms acquisition) on the dendritic shaft (dashed lines). Scale bar: 500 nm (bottom panel). **(B)** Comparison of D1R-CFP-antiGFPAb-QD complexes mean square displacements (MSD, mean ± SEM) in the absence (Healthy-) or presence of NMDAR-Ab from healthy carriers (Healthy+), or from patients with NMDAR-encephalitis (Enceph) or schizophrenia (SCZ+). ****SCZ+ vs Healthy- p < 0.0001, ^####^Enceph vs Healthy- p < 0.0001, ^##^Enceph vs Healthy- p = 0.0048. **(C)** Comparison of D1R-CFP-antiGFPAb-QD complexes instantaneous diffusion coefficients (median ± interquartile range 25–75%) in the absence (Healthy-) or the presence of NMDAR-Ab from healthy carriers (Healthy+) or patients with NMDAR-encephalitis (Enceph) or schizophrenia (SCZ+). ****p < 0.0001, ***p = 0.0006, *p = 0.0119. **(D)** Representation of full D1R containing t2 segment enabling the physical interaction with NMDAR (top) and the mutated D1R (D1RΔt2-CFP) preventing its binding to NMDAR (bottom) (left). Comparison of D1R-CFP-antiGFPAb-QD/D1RΔt2-CFP-antiGFPAb-QD complexes surface dynamics (MSD, mean ± SEM) in the absence (Healthy-) or presence (SCZ+) of NMDAR-Ab from patient #2 with schizophrenia (right).

As NMDAR-Ab from NMDAR-encephalitis patients mainly bind to NMDAR in the hippocampus both in humans and rodents despite their brain widespread distribution (18), we assessed the impact of autoantibodies on D1R surface dynamics on hippocampal cultured neurons prepared from E18 Sprague-Dawley rats. At 7–11 days of development *in vitro*, neurons were co-transfected with D1R fused to the cyan fluorescent protein (D1R-CFP) and Homer1C fused to DsRed protein DNAs to specifically track and concentrate our analysis on the extrasynaptic D1R pool, as we previously demonstrated that the vast majority of D1R are located outside hippocampal synapses (19, 20). In addition, since NMDAR-Ab could alter the surface dynamics of D1R through a domino effect due to the physical interaction between NMDAR and D1R, we assessed the impact of autoantibodies from one patient with schizophrenia on D1R surface dynamics in neurons in which this physical interaction was genetically prevented by expressing the intracellular C-terminus t2 segment-truncated D1R-CFP (D1RΔt2-CFP; see **Figure 1D**). Quantum dot (QD) tracking of D1R-CFP (or D1RΔt2-CFP) was performed on live hippocampal neurons at 12–15 days of development *in vitro*. Neurons were first incubated overnight (14 ± 2 h) with NMDAR-Ab containing purified IgGs (5 µg/ml) from either patients with NMDAR-encephalitis (Enceph), schizophrenia (SCZ+), or healthy carriers (Healthy+), or with purified IgG (5 µg/ml) from healthy seronegative subjects (Healthy-) (**Figure 1A**, top). Incubation time was increased compared with that in previous report that failed to reveal any effect of NMDAR-Ab on D1R surface dynamics when using a shorter exposure (2 h) (8). For QD labeling and microscopy, hippocampal neurons were then incubated (10 min) with anti-GFP antibodies (Molecular Probes A6455, 1/10,000 to 1/20,000 dilution). Neurons were then washed and incubated (10 min) with QDs coupled to an anti-Rabbit F(ab) fragment (Life Technologies Q11421MP, 1/100,000 dilution). Images were obtained with an acquisition time of 50 ms with up to 500 consecutive frames. The instantaneous diffusion coefficient, D, was calculated for each trajectory, from linear fits of the first four points of the mean square displacement (MSD) versus time (t) function using MSD(t) = < r2>(t) = 4Dt.

### Statistical Analysis

Comparisons between conditions were made running nonparametric Kruskall–Wallis tests followed by Dunn’s multiple comparisons. Analyses were performed using GraphPad Prism 8 for Windows software (version 8.0.2, GraphPad Software, Inc) with a statistical significance of 0.05.

## Results

As D1R physically interacts with NMDAR, we explored the possibility that circulating NMDAR-Ab from patients with NMDAR-encephalitis or schizophrenia alter as a mechanical consequence the lateral dynamics of D1R. Hippocampal neurons expressing exogenous D1R were exposed to either NMDAR-Ab-containing IgGs (overnight, 5 µg/ml) from patients (Enceph or SCZ+), healthy carriers (Healthy+), or IgGs from seronegative controls (Healthy-). Single nanoparticle tracking of D1R was then performed to investigate the impact of NMDAR-Ab on D1R membrane dynamics (**Figure 1A**). As D1R being mostly located outside glutamatergic synapses (19, 20), we specifically analyzed the extrasynaptic diffusion. Surface dynamics of D1R was assessed by measuring their MSD curves and instantaneous diffusion coefficients (21). NMDAR-Ab from patients with NMDAR-encephalitis or schizophrenia, but not from healthy carriers, decrease the explored areas and instantaneous diffusion coefficients of D1R when compared with the Healthy- condition (**Figures 1B, C**, **Table 2**). Noteworthy, NMDAR-Ab from healthy carriers did not differ from healthy seronegative controls. These data suggest that only NMDAR-Ab from patients have the potency to alter the dynamics of D1R, likely through a “domino effect” in which the physical interplay between NMDAR and D1R controls the single molecule behavior of each partner. To directly address this possibility, we investigated the impact of NMDAR-Ab from one patient with schizophrenia on D1R that were genetically prevented to interact with GluN1-NMDAR (D1RΔt2-CFP): the intracellular t2 segment is a major binding sequence to the GluN1 subunit (17) (**Figure 1D**, left). Remarkably, the surface dynamics (MSD) of the D1R mutant was not decreased by NMDAR-Ab from the patient but instead significantly increased (**Figure 1D**, right), indicating that the alteration of D1R trafficking by NMDAR-Ab patients is regulated, at least in part, by the physical interaction with NMDAR.

**Table 2:** Statistical analysis and values. MSD, mean square displacement; N, number of trajectories.

Fig	Parameter	Conditions	Values Median ± 25–75% IQR	N	Statistical test α = 0.05	P value
**1B**	MSD (at time lag = 0.4 s) MSD (at time lag = 0.5 s) MSD (at time lag = 0.6 s)	Healthy- Healthy+ Enceph SCZ+ Healthy- Healthy+ Enceph SCZ+ Healthy- Healthy+ Enceph SCZ+	0.1060 ± 0.05160–0.1900 µm^2^ 0.1100 ± 0.05100–0.1950 µm^2^ 0.1010 ± 0.04678–0.1710 µm^2^ 0.09405 ± 0.04120–0.1770 µm^2^ 0.1240 ± 0.06030–0.2270 µm^2^ 0.1305 ± 0.05940–0.2350 µm^2^ 0.1200 ± 0.05488–0.2070 µm^2^ 0.1110 ± 0.04725–0.2080 µm^2^ 0.1420 ± 0.06805–0.2630 µm^2^ 0.1545 ± 0.06995–0.2733 µm^2^ 0.1371 ± 0.06000–0.2380 µm^2^ 0.1240 ± 0.05000–0.2430 µm^2^	4,505 891 3,750 3,498 3,761 728 2,998 3,113 3,380 646 2,723 2,747	Kruskal–Wallis (p < 0.0001) followed by Dunn’s multiple comparison test	Healthy+ vs Healthy- > 0.9999 ns Enceph vs Healthy- < 0.0001 SCZ+ vs Healthy- < 0.0001 Enceph vs SCZ+ > 0.9999 ns Healthy+ vs Healthy- > 0.9999 ns Enceph vs Healthy- < 0.0001 SCZ+ vs Healthy- < 0.0001 Enceph vs SCZ+ > 0.9999 ns Healthy+ vs Healthy- > 0.9999 ns Enceph vs Healthy- = 0.0048 SCZ+ vs Healthy- < 0.0001 Enceph vs SCZ+ = 0.3976 ns
**1C**	Instantaneous diffusion coefficient	Healthy- Healthy+ Enceph SCZ+	0.06546 ± 0.02720–0.1276 µm^2^/s 0.06801 ± 0.02735–0.1306 µm^2^/s 0.06080 ± 0.02430–0.1153 µm^2^/s 0.05715 ± 0.02015–0.1160 µm^2^/s	5,127 1,000 4,321 4,003	Kruskal–Wallis (p < 0.0001) followed by Dunn’s multiple comparison test	Healthy+ vs Healthy- > 0.9999 ns Enceph vs Healthy- =0.0006 SCZ+ vs Healthy- < 0.0001 Enceph vs SCZ+ = 0.4711 ns Enceph vs Healthy+ = 0.0119 SCZ+ vs Healthy+ = 0.0002
**1D**	MSD (at time lag = 0.4 s)	D1R Healthy- D1RΔt2 Healthy- D1R SCZ+ D1RΔt2 SCZ+	0.09415 ± 0.04065–0.1740 µm^2^ 0.1070 ± 0.05130–0.1820 µm^2^ 0.08990 ± 0.04260–0.1590 µm^2^ 0.1280 ± 0.05940–0.2170 µm^2^	1,664 1,287 1,869 1,615		D1R Healthy- vs D1RΔt2 Healthy- = 0.0537 ns D1R Healthy- vs D1R SCZ+ > 0.9999 ns D1RΔt2 SCZ+ vs others <0.0001
	MSD (at time lag = 0.5 s)	D1R Healthy- D1RΔt2 Healthy- D1R SCZ+ D1RΔt2 SCZ+	0.1150 ± 0.04888–0.2110 µm^2^ 0.1320 ± 0.06150–0.2250 µm^2^ 0.1070 ± 0.04840–0.1860 µm^2^ 0.1560 ± 0.07135–0.2670 µm^2^	1,410 1,103 1,555 1,381	Kruskal–Wallis (p < 0.0001) followed by Dunn’s multiple comparison test	D1R Healthy- vs D1RΔt2 Healthy- = 0.0586 ns D1R Healthy- vs D1R SCZ+ = 0.2643 ns D1RΔt2 SCZ+ vs D1RΔt2 Healthy- = 0.0008 D1RΔt2 SCZ+ vs D1R Healthy- < 0.0001 D1RΔt2 SCZ+ vs D1R SCZ+ < 0.0001 D1R Healthy- vs D1RΔt2 Healthy- = 0.1109 ns D1R Healthy- vs D1R SCZ+ = 0.0703 ns D1RΔt2 SCZ+ vs others <0.0001
	MSD (at time lag = 0.6 s)	D1R Healthy- D1RΔt2 Healthy- D1R SCZ+ D1RΔt2 SCZ+	0.1325 ± 0.05323–0.2450 µm^2^ 0.1520 ± 0.06730–0.2550 µm^2^ 0.1190 ± 0.05275–0.2145 µm^2^ 0.1840 ± 0.08320–0.3150 µm^2^	1,256 975 1,377 1,223		

## Discussion

Dopamine is a powerful modulator of the glutamatergic neurotransmission, acting mostly through the metabotropic actions, e.g., intracellular cascades, of its receptor family (22, 23). However, the physical interaction of membrane dopamine receptors with several other receptors, such as the NMDAR (17), provides an additional way to modulate the synaptic activity through the presence of receptor hetero-complexes (24). For instance, the activation of D1R disrupts the D1R-NMDAR interaction, increases NMDAR synaptic content through a fast lateral redistribution, and favors NMDAR-dependent long-term potentiation of glutamatergic synapses in a model of cultured hippocampal neurons (19). Here, we investigated, in the same model, whether the well-defined alteration of the NMDAR surface dynamics by NMDAR-Ab from patients sharing psychotic-like symptoms also perturbs, as a consequence, D1R dynamics. We demonstrate that an overnight incubation of hippocampal neurons with NMDAR-Ab from patients with NMDAR-encephalitis or schizophrenia, but not from healthy carriers, alters the surface dynamics of D1R. The fact that a shorter incubation (2 h) did not alter D1R surface dynamics supports the notion that the NMDAR-Ab effect is time dependent and likely indirect. Furthermore, the magnitude of NMDAR-Ab effects on D1R (∼10%) is, by far, weaker than the one on NMDAR (∼3-fold) (8, 12), likely due to the fact that only a fraction of D1R interacts with NMDAR and is thus prone to destabilization by NMDAR-Ab (19).

Both NMDAR-Ab from patients with encephalitis and schizophrenia were found to slowdown D1R surface dynamics. This is likely a mechanical consequence of the NMDAR immobilization triggered by autoantibodies in the extrasynaptic compartment where D1R is mainly located. Indeed, when the physical D1R-NMDAR interaction was genetically prevented, the D1R dynamics downregulation by NMDAR-Ab from patient with schizophrenia was abolished. To note, D1R surface dynamics was even upregulated in this condition, as expected from the NMDAR-Ab-induced NMDAR crosslinking and internalization (8, 9).

Collectively, we here demonstrated that NMDAR-Ab, which primarily target and alter NMDAR surface organization, also disorganize its membrane partner D1R. However, we highlighted that the effect of the NMDAR-Ab is relatively weaker on D1R when compared with that on NMDAR. Importantly, NMDAR-Ab from different origins (patients versus healthy carriers) do not necessarily share the same molecular impact on the glutamatergic and dopaminergic receptor trafficking. This is consistent with previous finding demonstrating that NMDAR-Ab from healthy carriers or patient with autism spectrum disorder without history of psychosis do not alter NMDAR surface trafficking (12, 25). Our data further highlight that NMDAR-Ab are diverse in their mechanisms of action and call for further investigations to decrypt the alterations on the targeted NMDAR and its membrane partners.

## Ethics Statement

Patients with NMDAR encephalitis and schizophrenic (DSM-IV criteria) were included in this study after approval by a French ethical committee and written informed consent for their participation.

## Author Contributions

HG and LG designed the study. VR, NH, EG, RT, ED, CP, ML, and JH performed clinical analysis. HG performed single nanoparticle experiments. DB performed molecular biology and cell biology preparation. HG analyzed the data. HG and LG wrote the paper.

## Funding

This study was financially supported by the Centre National de la Recherche Scientifique, Agence Nationale de la Recherche (ANR-14-CE15-0001), Fondation pour la Recherche Médicale, Conseil Régional d’Aquitaine, Labex Bordeaux BRAIN, IDEX Bordeaux, fondation FondaMental, Labex Bio-PSY, and Ministère de l’Enseignement supérieur et de la Recherche.

## Conflict of Interest Statement

The authors declare that the research was conducted in the absence of any commercial or financial relationships that could be construed as a potential conflict of interest.

## Abbreviations

NMDAR-Ab, N-methyl-D-aspartate receptor autoantibodies; CFP, cyan fluorescent protein; D1R, dopamine D1 receptor; D1R-Δt2, intracellular t2 segment truncated dopamine D1 receptor; IgGs, type G immunoglobulins; QD, quantum dot; Healthy-, healthy subjects seronegative for NMDAR-Ab; Healthy +, healthy subjects seropositive for NMDAR-Ab; Enceph, NMDAR-encephalitis patients (inherently seropositive for NMDAR-Ab); SCZ+, patients with schizophrenia seropositive for NMDAR-Ab; MSD, mean square displacement.
